# Kinome and mRNA expression profiling of high-grade osteosarcoma cell lines implies Akt signaling as possible target for therapy

**DOI:** 10.1186/1755-8794-7-4

**Published:** 2014-01-21

**Authors:** Marieke L Kuijjer, Brendy EWM van den Akker, Riet Hilhorst, Monique Mommersteeg, Emilie P Buddingh, Massimo Serra, Horst Bürger, Pancras CW Hogendoorn, Anne-Marie Cleton-Jansen

**Affiliations:** 1 Department of Pathology, Leiden University Medical Center, Albinusdreef 2, 2300RC Leiden, The Netherlands; 2 Current affiliation: Department of Biostatistics and Computational Biology, Dana-Farber Cancer Institute, Boston, MA, USA; 3 Current affiliation: Department of Biostatistics, Harvard School of Public Health, Boston, MA, USA; 4 PamGene International BV, ‘s-Hertogenbosch, The Netherlands; 5 Department of Pediatrics, Leiden University Medical Center, Leiden, The Netherlands; 6 Laboratory of Experimental Oncology, Istituto Ortopedico Rizzoli, Bologna, Italy; 7 Institute of Pathology, Paderborn/Höxter, Germany

**Keywords:** Osteosarcoma, Tumor cell lines, Kinome profiling, Gene expression profiling, Genomic instability, Bone tumor

## Abstract

**Background:**

High-grade osteosarcoma is a primary malignant bone tumor mostly occurring in adolescents and young adults, with a second peak at middle age. Overall survival is approximately 60%, and has not significantly increased since the introduction of neoadjuvant chemotherapy in the 1970s. The genomic profile of high-grade osteosarcoma is complex and heterogeneous. Integration of different types of genome-wide data may be advantageous in extracting relevant information from the large number of aberrations detected in this tumor.

**Methods:**

We analyzed genome-wide gene expression data of osteosarcoma cell lines and integrated these data with a kinome screen. Data were analyzed in statistical language R, using *LIMMA* for detection of differential expression/phosphorylation. We subsequently used Ingenuity Pathways Analysis to determine deregulated pathways in both data types.

**Results:**

Gene set enrichment indicated that pathways important in genomic stability are highly deregulated in these tumors, with many genes showing upregulation, which could be used as a prognostic marker, and with kinases phosphorylating peptides in these pathways. Akt and AMPK signaling were identified as active and inactive, respectively. As these pathways have an opposite role on mTORC1 signaling, we set out to inhibit Akt kinases with the allosteric Akt inhibitor MK-2206. This resulted in inhibition of proliferation of osteosarcoma cell lines U-2 OS and HOS, but not of 143B, which harbors a *KRAS* oncogenic transformation.

**Conclusions:**

We identified both overexpression and hyperphosphorylation in pathways playing a role in genomic stability. Kinome profiling identified active Akt signaling, which could inhibit proliferation in 2/3 osteosarcoma cell lines. Inhibition of PI3K/Akt/mTORC1 signaling may be effective in osteosarcoma, but further studies are required to determine whether this pathway is active in a substantial subgroup of this heterogeneous tumor.

## Background

High-grade osteosarcoma is the most prevalent primary malignant bone tumor. Most frequently, the long bones of adolescents and young adults are affected, with a yearly incidence of approximately 5 cases per million per year [1]. Patients are generally treated with high doses of neoadjuvant chemotherapy to prevent the outgrowth of micrometastases. In 15-25% of all patients, however, metastatic disease is clinically detectable at diagnosis and despite the intensive treatment, 45% of all patients develop distant metastases, the leading cause of death of osteosarcoma patients [2,3]. The introduction of neoadjuvant chemotherapy in the 1970s has increased survival from 10-20% to approximately 60%. However, survival has reached a plateau, and new treatments are urgently needed [4-6]. Osteosarcoma is an extremely genomically unstable tumor, with karyotypes harboring numerous numerical and structural changes [7,8]. In addition, osteosarcoma genotypes show a considerable degree of heterogeneity, both intra- and intertumoral. Both the complex genotype and its heterogeneity render it difficult to determine which genomic alterations are important in osteosarcomagenesis, as not all alterations may lead to a difference in mRNA, protein levels, or enzyme activity in the tumor tissue. Integration of different data types is therefore of particular relevance for studying a heterogeneous tumor with a complex genomic profile such as osteosarcoma. Genomic and expression data of osteosarcoma tumor samples have been integrated by different groups, and many of the reported recurrent osteosarcoma driver genes play a role in cell cycle regulation and maintenance of genomic stability [9,10]. Yet, even though recurrent driver genes may provide knowledge on what pathways are affected that help tumor cells survive, such driver genes may not always be accessible as targets for treatment. This especially holds for pathways involved in genetic stability, since the damage is already done.

Oncogenic kinases are often active in tumor cells, and a number of kinases can be pharmacologically inhibited. Therapies targeting oncogenic kinases have provided promising results in inhibiting proliferation of cancer cells, and some kinases have been targeted in preclinical and clinical studies in childhood sarcomas (as reviewed in Wachtel *et al*. [11]), *e.g.* IGF1R and mTOR [12,13]. An unbiased approach to identify active kinases in cancer is to perform kinome-wide screens. Such screens have previously been effectively used in other types of sarcoma and have led to the detection of specific targets for treatment [14,15]. As combining the analysis of different data types using systems biology approaches can give a more complete impression of the state of a tumor cell, we set out to integrate genome-wide gene expression data of osteosarcoma cell lines with kinome profiling data. Osteosarcoma cell lines are widely available and have been shown to be representative for the tumor of origin, both on a genome-wide as on a functional level, and are therefore a good model to study osteosarcoma preclinically [9,16].

We previously have performed genome-wide expression analysis on a panel of 19 osteosarcoma cell lines [17]. In the present study, we compared these expression profiles with the different putative progenitor cells of osteosarcoma – mesenchymal stem cells (MSCs) and osteoblasts – in order to define the common denominator pathways that are deregulated in osteosarcoma. We then integrated expression data with a serine/threonine (Ser/Thr) kinome screen, to determine whether pathways with enrichment of differentially expressed genes show enrichment in of hyperphosphorylation as well. In order to detect overactive kinases in osteosarcoma, which may be potential targets for treatment, we identified the most significant pathways by a single-way analysis of the kinome profiling data.

## Methods

### Cell culture

Osteosarcoma cell lines were previously characterized and described [17]. Human bone-marrow-derived MSCs were obtained from two osteosarcoma patients, and were characterized and handled as described [18]. For kinome profiling of osteosarcoma versus MSCs, cells were cultured in Dulbecco’s Modified Eagle Medium (DMEM; Invitrogen, Carlsbad, CA, USA), supplemented with 10% fetal bovine serum (Greiner Bio-one, Frickenhausen, Germany), in order to eliminate differences in kinase activity caused by culture conditions. For inhibition experiments and kinome profiling of inhibition experiments, osteosarcoma cell lines 143B, U-2 OS, and HOS were maintained in RPMI 1640 supplemented with 10% fetal calf serum (both from Invitrogen, Carlsbad, CA). The human pre-B acute lymphoblastic leukemia cell line NALM-6 cell line was kindly provided by Mw. N. Duinkerken (Department of Hematology, Leiden University Medical Center, the Netherlands), and was maintained in Iscove’s Modified Dulbecco’s Medium (IMDM) supplemented with GlutaMAX-1 (Life Technologies, Carlsbad, CA) and 10% fetal bovine serum (Greiner Bio-one, Frickenhausen, Germany). All cells were regularly tested for mycoplasm and were genotyped before and after experiments using the Powerplex 1.2 system (Promega, Leiden, the Netherlands), as described previously [16], and using CellID STR profiling (Promega, Leiden, the Netherlands). Latest genotyping results are added in Additional file 1). Cell lines corresponded to the entries in the ATCC (http://www.atcc.org) and DSMZ (http://www.dsmz.de) databases.

### Cell lysates

Kinome profiling was performed on osteosarcoma cell lines 143B and U-2 OS and on two MSCs – MSC001 and MSC006. Cells at 80% confluence were washed twice with Phosphate buffered Saline and lysed with M-PER Mammalian Extraction Buffer, supplemented with Halt Phosphatase Inhibitor Cocktail and EDTA free Halt Protease Inhibitor Cocktail (Pierce Biotechnology, Rockford, IL), according to the manufacture’s protocol. Cells were incubated on ice for at least 30 minutes before collecting the lysates and centrifuging these for 15 minutes at 4°C at > 10,000× g. Protein concentration was measured using a detergent-compatible Protein Assay (Bio-Rad Laboratories, Hercules, CA) according to the manufacturer’s protocol. Samples were snap-frozen and stored at −70°C.

### Proliferation assays

MK-2206 was dissolved in DMSO at a concentration of 10 mM and stored at −20°C. For 143B, U-2 OS, and HOS, 2,000, 4,000, and 2,000 cells/well respectively, were plated in a 96-wells plate. NALM-6, a human pre-B acute lymphoblastic leukemia (ALL) cell line, was included as a positive control, as ALL cell lines have been shown to be highly sensitive to MK-2206 [19]. This cell line grows in suspension and was plated at 50,000 cells/well. After 24 hrs, MK-2206 was added in triplicate in different concentrations – 0 nM, 0.5 nM, 1 nM, 5 nM, 10 nM, 50 nM, 100 nM, 500 nM, 1 μM, 5 μM, and 10 μM. For 143B and HOS, the effect of concentrations of 2, 3, 4, and 5 nM was assessed as well. Cells were grown in the presence of inhibitor for 120 hours. Cell proliferation was determined by incubating the cells with reagent WST-1 (Roche, Basel, Switzerland) for 2 hrs and subsequently measured using a Wallac 1420 VICTOR2 (Perkin Elmer, Waltham, MA). Data were analyzed in Graphpad Prism 5.01 (http://www.graphpad.com). Relative IC_50_s were calculated using results from the different concentrations up to the highest dose where toxicity was not yet present. The results shown are representative results from at least three independent experiments.

### Genome-wide gene expression profiling

We analyzed our previously published data of osteosarcoma cell lines (n = 19), MSCs (n = 12), and osteoblasts (n = 3) (GEO superseries, accession number GSE42352) [9]. Microarray data processing and quality control were performed in the statistical language R version 2.15 [20] as described previously [21].

### Kinome profiling

Kinome profiling was performed on 1 μg of cell lysate on the serine/threonine (Ser/Thr) Kinase PamChip® peptide microarrays (PamGene, ‘s-Hertogenbosch, the Netherlands) according to the manufacturer’s protocol, essentially as described in Hilhorst *et al*. [22]. This peptide microarray comprises 142 peptide sequences derived from human phosphorylation sites. Peptide phosphorylation is detected in time with a mixture of fluorescently labeled anti-phosphoserine/threonine antibodies. We used at least three technical replicates for each MSC line, and four technical replicates for the osteosarcoma cell lines. Images were taken every 5 minutes, over the course of 60 minutes. Signal quantification on phosphorylated peptides was performed in BioNavigator software (PamGene International, ‘s Hertogenbosch, the Netherlands). Subsequently, data were normalized in R [23] using the vsn package [24]. Median signals at 60 minutes of incubation with the cell lysates were analyzed in Bioconductor [25] package array QualityMetrics [26] to identify poor quality samples, which were removed from further analysis. Technical replicates of good quality were averaged. To determine whether these data were reproducible, we analyzed data from different cycles (0, 10, 20, 30, 40, 50, and 60 minutes incubation with cell lysates).

In the second kinome profiling experiment we compared lysates of untreated cells with lysates of cells treated with MK-2206. Different treatment durations and concentrations were used – no treatment, treatment for 5, 30, 180, and 960 minutes with 1 μM MK-2206, and treatment for 180 minutes with 10 μM of the drug. Kinome profiling was performed as described above, with the difference that we used 1–5 technical replicates per condition. Of this experiment, we analyzed signals at 30 minutes of incubation with the lysates.

### Statistical analyses of microarray data

We performed *LIMMA* analysis [23] in order to determine differential mRNA expression between osteosarcoma cell lines (n = 19) and control cell lines – MSCs (n = 12) and osteoblasts (n = 3) and to determine differential phosphorylation of peptides on the PamChip® microarray between osteosarcoma cell lines (n = 2) and MSCs (n = 2). We used a Benjamini and Hochberg False Discovery Rate (FDR) of 0.05 as cut-off for significance. Kinome profiling signals obtained for the different treatment conditions were analyzed in a paired approach, in which signals from untreated cells were subtracted from the signals from treated cells. For both kinome profiling experiments, we used a cut-off of 0.1 for the absolute log fold change (logFC). Heatmaps were generated using the function *heatmap.2* of R package *gplots*.

### Pathway analysis

In order to reveal pathways which were significantly affected on mRNA levels in osteosarcoma cell lines, we intersected the toptables obtained by *LIMMA* analysis of osteosarcoma cell lines versus MSCs and of osteosarcoma cell lines versus osteoblasts. Gene symbols for all probes were imported into the software Ingenuity Pathways Analysis (IPA, Ingenuity Systems, http://www.ingenuity.com), together with FDR adjusted *P*-values (adj*P*) and average logFCs. Only the gene symbols of probes that were both significantly upregulated or both significantly downregulated in osteosarcoma cell lines as compared with MSCs and with OBs (adj*P* < 0.05) were selected to be considered as significantly differentially expressed in the IPA analysis. For differential phosphorylation, we imported the results from the *LIMMA* analysis on kinome profiling data, with a cut-off of 0.05 for adjusted *P*-value and a cut-off of 0.1 for logFC. The significance of the association between the data set and the canonical pathways was measured as described previously [27]. Pathways with adj*P* < 0.05 were considered to be significantly affected. In addition, transcription factor analyses were performed on gene expression data in IPA in order to predict activated or inhibited transcription factors based on expression of target genes, returning p-values (with a cut-off of 0.05 for significance) and regulation z-scores.

## Results

### Genome-wide gene expression profiling of high-grade osteosarcoma cell lines

**Figure 1:**
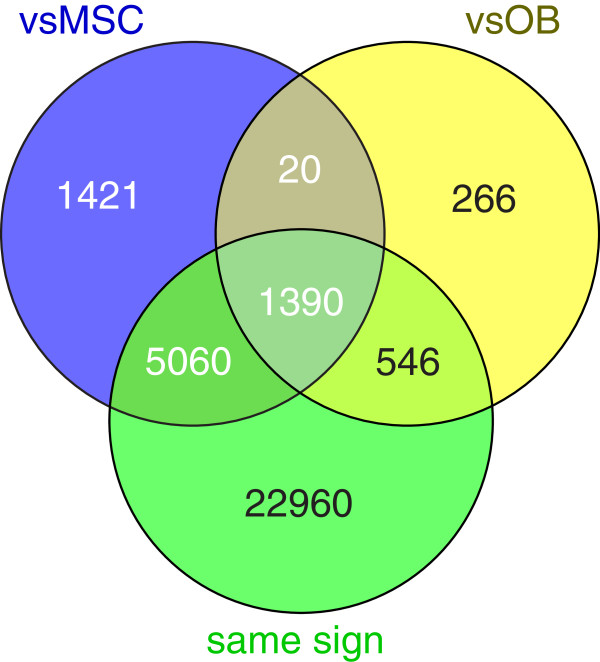
**Intersection of top lists.** Venn diagram showing the significant probes in the analysis of osteosarcoma cell lines vs MSC (vsMSC) and vs osteoblasts (vsOB), and the intersection of these significant probes with the subset of all probes (both significant and nonsignificant) that shows both up- or both downregulation in these two analyses (same sign). In total, 1,410 probes are significant in both analyses, of which 1,390 have the same sign of logFC.

We started by comparing gene expression signatures of 19 osteosarcoma cell lines, 12 MSC, and 3 osteoblast cultures using unsupervised hierarchical clustering. Two separate clusters were detected – one containing all tumor cell samples and one containing control samples. Within the control sample cluster, osteoblasts clustered separately from MSCs (Additional file 2). *LIMMA* analysis resulted in 7,891 probes encoding for differentially expressed (DE) genes between osteosarcoma cell lines and MSCs, and 2,222 probes encoding for DE genes between osteosarcoma cells and osteoblasts (Additional file 3). Intersecting of these gene lists showed 1,410 probes that were significant in both analyses, of which 1,390 were upregulated in both analyses, or downregulated in both analyses (Figure 1). These probes, encoding for 1,312 genes, were selected for subsequent pathways analysis, in order to determine commonly affected pathways in osteosarcoma tumor cells.

### Gene expression is altered in pathways regulating genomic stability

**Figure 2:**
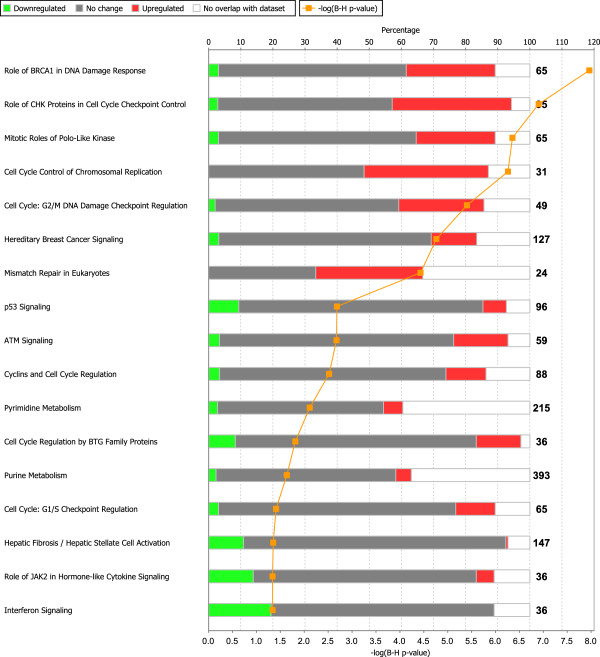
**Significantly affected pathways in osteosarcoma cells.** Stacked bar chart depicting all significantly affected pathways as identified by gene expression profiling of osteosarcoma cell lines, showing percentages of up- (red), downregulated (green), not significantly altered genes (gray), and genes which were not present on the microarray (white). The –log(adj*P*) (−log(B-H) p-value) is plotted in orange, and is above 1.3 for adj*P* < 0.05.

Pathway analyses on the 1,312 differentially expressed genes resulted in 17 significantly affected pathways (Figure 2). 14 out of these 17 pathways play a direct or indirect role in genomic stability. Unsupervised hierarchical clustering of all cell line data and data from 84 osteosarcoma biopsies (GEO accession number GSE33382, [9]) was performed on all DE genes present in these 17 significantly affected pathways, which resulted in a cluster of control cells and biopsies, and larger cluster of osteosarcoma cell lines and biopsies (Additional file 4). Patients whose biopsies had expression profiles of these pathways similar to osteosarcoma cell lines showed worse metastasis-free survival than patients with intermediate expression profiles, and than patients whose biopsies had expression profiles more similar to the control cultures, *i.e.* non-transformed primary mesenchymal cell cultures and osteoblast cultures (log-rank test for trend, *P* = 0.049, Additional file 5). Transcription factors that were predicted to be activated or inhibited based on expression of target genes are shown in Additional file 6. The most activated transcription factor was *MYC*, while the most inactivated transcription factor was *TP53*.

### Kinome profiling of osteosarcoma cell lines

**Figure 3:**
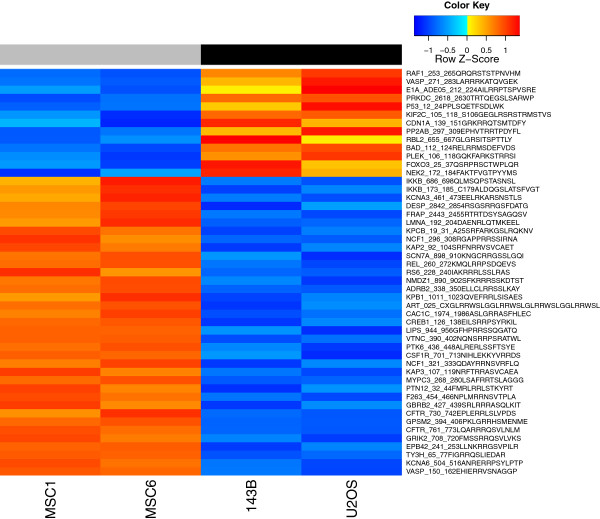
**Supervised clustering of kinome profiling results.** Supervised clustering on all 49 significantly differentially phosphorylated peptides identified by the comparison of two osteosarcoma cell lines with two MSC cultures. Peptides are sorted on logFC, from lower phosphorylation to higher phosphorylation in osteosarcoma cell lines. Orange: higher phosphorylation levels, blue: lower phosphorylation levels.

To obtain more information on the activity of the pathways which showed aberrant mRNA expression, we integrated mRNA expression data with data obtained with kinase PamChip® peptide microarrays. These peptide microarrays were incubated with lysates of the osteosarcoma cell lines 143B and U-2 OS, two of the most widely used osteosarcoma cell lines, of which 143B is the only human osteosarcoma cell line with metastatic behaviour in a mouse xenograft model [16], and with lysates of two human MSC cultures. Kinases present in the cell lysates can, in the presence of ATP, phosphorylate the peptides present on the microarray, which is detected by fluorescently labeled antibodies. We compared kinome profiling data at different incubation times by intersecting lists of differentially phosphorylated peptides between osteosarcoma cells and MSCs, obtained by *LIMMA* analyses, as shown in Additional file 7. This data analysis demonstrated a large overlap in the detected differentially phosphorylated peptides, and a build-up of differentially phosphorylated peptides over time. Most peptides showed differential phosphorylation after 20 minutes of incubation with cell lysates. After 60 minutes of incubation on the peptide microarray, 49 peptides were detected to be significantly differentially phosphorylated between osteosarcoma cell lines and mesenchymal stem cells. These peptides are represented in Figure 3. As a reference, we performed an unsupervised hierarchical clustering including all technical replicates (Additional file 8), which showed that phosphorylation of peptides by cell lysates of most technical replicates was comparable.

### Altered phosphorylation in genomic stability pathways

**Figure 4:**
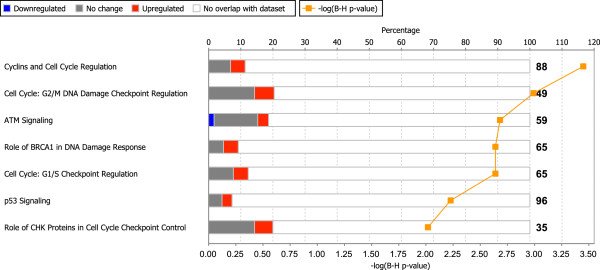
**Kinome profiling pathway analysis on the set of significant pathways from gene expression profiling.** Stacked bar chart showing kinome profiling pathway analysis on the subset of pathways which were significant on gene expression profiling. Percentages of up- (orange), downregulated (blue), not significantly altered genes (gray), and genes which were not present on the microarray (white) are shown. The –log(adj*P*) (−log(B-H) p-value) is plotted in orange, and is above 1.3 for adj*P* < 0.05.

The significance of the 17 pathways that were returned from the pathway analysis on mRNA expression data was tested on kinome profiling results in IPA. In total, 7/17 pathways were significant in kinome profiling as well. These seven pathways were a subset of the 14 pathways with a known role in genomic stability and cell cycle progression. Most significantly differentially phosphorylated peptides in these seven pathways showed higher phosphorylation levels in osteosarcoma cell lines (Figure 4), indicating that kinases affect phosphorylation of molecules playing a role in genomic stability and cell cycle progression.

### PI3K/Akt and AMPK signaling in osteosarcoma

**Figure 5:**
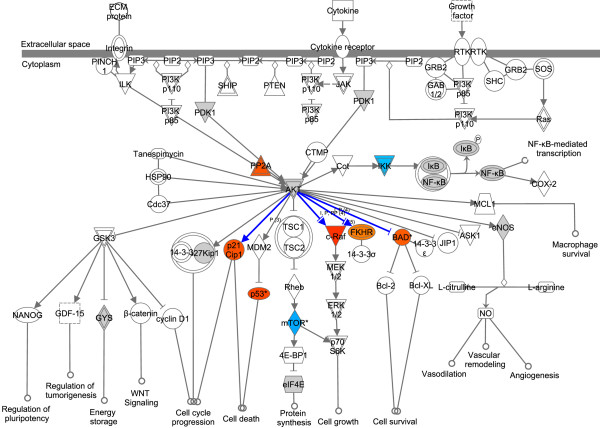
**Akt signaling pathway.** The Akt signaling pathway in IPA. Blue: significantly lower, orange: significantly higher phosphorylation in osteosarcoma cell lines, gray, no significant difference in phosphorylation, white: no phosphorylation sites of the particular protein on the PamGene Ser/Thr chip. Blue lines indicate known downstream phosphorylation by the upstream kinase.

Unsupervised pathway analysis on the kinome profiling results returned the IPA pathway PI3K/Akt signaling as the most significantly affected pathway in osteosarcoma cells (Figure 5) and the AMPK pathway as second most significantly affected pathway (Additional file 9). Specifically, molecules directly downstream of Akt kinases showed higher phosphorylation in osteosarcoma than in MSCs, while molecules downstream of AMPK showed lower phosphorylation levels. As these results indicate that Akt signaling is active in osteosarcoma and might be driving its high proliferative capacity, we set out to pharmacologically inhibit Akt using the compound MK-2206.

### MK-2206 inhibits proliferation of U-2 OS and HOS, but not of 143B

**Figure 6:**
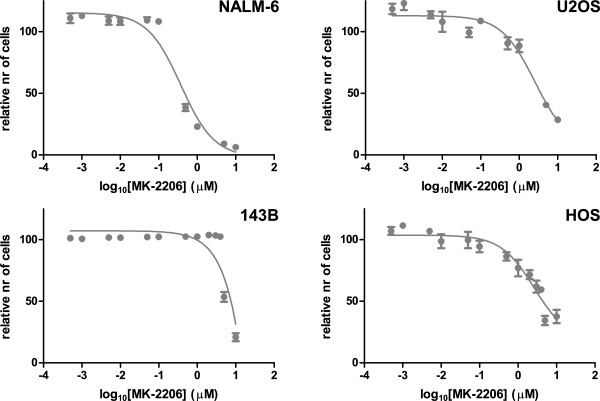
**Proliferation of osteosarcoma cell lines was inhibited with different concentrations of MK-2206, for 120 hours.** NALM-6, U-2 OS, and HOS showed a dose-dependent inhibition, while 143B did not respond.

We inhibited osteosarcoma and control cells for 120 h with MK-2206, an allosteric inhibitor of all three Akt family members. Inhibition of the positive control leukemia cell line NALM-6, and of osteosarcoma cell line U-2 OS with MK-2206 was dose-dependent, with IC_50_s of 0.38 μM and 2.5 μM, and maximal responses of 94% and 71%, respectively (Figure 6). 143B did not show any response at concentrations below 5 μM. Because 143B exhibits an oncogenic *KRAS* transformation [28], we assessed MK-2206 specificity on the parental cell line of 143B, HOS, which has not been *KRAS* transformed. HOS indeed responded similar to U-2 OS, with an IC_50_ of 2.6 μM and maximal response of 62%.

### Different phosphorylation patterns upon treatment with MK-2206

**Figure 7:**
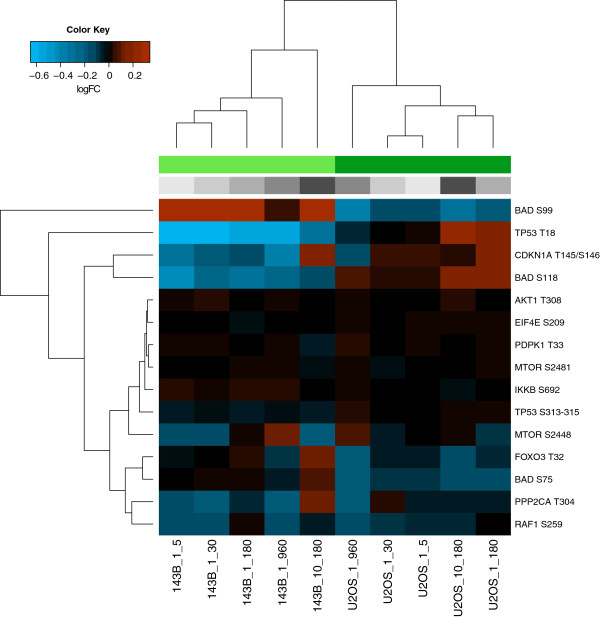
**Unsupervised clustering of peptides which can be phosphorylated by Akt.** Unsupervised clustering depicting differential phosphorylation of peptides of the PI3K/Akt pathway by cell lysates treated with different concentrations of MK—2206 and for different time intervals, as compared with untreated cells. Blue: logFC < 0, orange: logFC > 0. Different treatment options are shown in different shades of gray (from light to dark gray: 1 μM 5, 30, 180, 960, and 10 μM 180 minutes of treatment with MK-2206. Light-green: 143B, dark green: U-2 OS.

As 143B and U-2 OS showed different sensitivities to MK-2206, we performed a paired analysis between kinome profiling data obtained from lysates of cells, which were treated with different concentrations of MK-2206, and for different treatment lengths. Overall, the phosphorylation patterns differed between both cell lines, and distances between treatment options within each cell line were smaller than between the cell lines (Additional file 10). We generated a heatmap of differential phosphorylation in the paired analysis of treated and untreated cells, depicting all peptides of the PamGene chip which are downstream of PI3K/Akt (Figure 7). This figure shows that the inhibition pattern of MK-2206 is different in the two osteosarcoma cell lines, suggesting that other upstream kinases may be affected by inhibition of Akt with MK-2206 as well.

## Discussion

Osteosarcoma is a highly genomically unstable tumor. The identification of specific molecular targets that drive oncogenesis and that might be targets for therapy may thereby be hampered. Genome-wide gene expression profiling of high-grade osteosarcoma cell lines, in fact, showed an enrichment of differential expression in pathways important in genomic stability (Figure 2), with a role in cell cycle and checkpoint regulation (*e.g.* p53 signaling, G1/S and G2/M checkpoint regulation), DNA damage response (*e.g.* ATM signaling, role of *BRCA1* in DNA damage response), and purine/pyrimidine metabolism. Most significantly differentially expressed genes in these pathways were upregulated, for example *DNA-PK*, *BRCA1*, and *CDC25A*. Some downregulated genes were detected as well, such as *CDKN1A*, which has an inhibitory role on cell cycle progression, and genes downstream of *TP53* (*e.g. THBS1* and *SERPINE1*, encoding TSP1 and PAI-1, respectively). Expression levels of genes in these pathways in osteosarcoma pre-treatment biopsies correlated with survival, as was previously reported on the same dataset [9] by using the CIN25 signature [29].

IPA transcription factor analysis showed that *MYC* was the most significantly activated (z-score of 6.294), and *TP53* the most significantly inactivated (z-score of −7.660) transcription factor. Other highly predicted activated transcription factors were *e.g. E2F1/2/3* (Additional file 6). These different transcription factors are known to be affected in osteosarcoma [7,9,30]. The role of these transcription factors in cell cycle progression further confirms the importance of these pathways in osteosarcoma. Important to note is that we took a different approach to determine significantly altered pathways from in our previous publications [9,31]. We only used overlapping genes with same pattern of expression (both significant up- or downregulation) in osteosarcoma cell lines versus both control sets. This approach ensured us that all genes detected in the enriched pathways are significantly up- or downregulated in both comparisons, while our previous analyses described pathways which are significantly altered, but for which the gene list per pathway accounting for the significant effect may be different. We specifically took this more conservative approach for our current study, because we wanted to directly compare the expression levels and kinase activities of the specific players in each pathway. We also hypothesized that, using a method testing the overall aberration of a pathway, it would be more difficult to pick up specific players to inhibit pharmacologically. The pathways we detected with this analysis – pathways playing a role in cell cycling and genomic instability – were, as expected, also significantly affected in the less conservative globaltest analysis (which tests groups of genes instead of single genes) reported in our recent BMC Cancer publication [31] (*data not shown*).

Given the extreme genomic instability which is notorious in osteosarcoma and has led to the formulation of a novel genetic mechanism, chromothripsis [32], it is not surprising that the most prominent pathways are associated with this signature. Unfortunately pharmacological targeting of genomic instability is a challenge. Kinome-wide screens have previously led to the detection of specific targets for treatment in other sarcoma types [14,15], and as such a screen can complement us with extra information on aberrations in the pathways we detected with gene expression analyses, we performed kinome profiling of osteosarcoma cell lysates. Since the pathways that were shown to be significantly affected on mRNA expression mostly contained Ser/Thr kinases, we selected a Ser/Thr peptide microarray – the Ser/Thr PamChip®. Pathway analysis on kinome profiling data showed that 50% of the pathways that were significant on gene expression data were also significantly enriched in differential phosphorylation signals (Figure 4). All significant peptides were higher phosphorylated in osteosarcoma cell lines, except for a peptide present in CREB1. Since most of these peptides showed higher phosphorylation, we expect these pathways to be highly active, demonstrating higher cell cycling of the tumor cells, and deregulated responses to DNA damage.

We next determined the most significantly affected pathways in the kinome data from the entire IPA canonical pathways database, and detected deregulation of the PI3K/Akt and AMPK signaling pathways. Molecules downstream of Akt kinases showed higher phosphorylation (Figure 5), while downstream of AMPK, lower levels of phosphorylation were detected (Additional file 9). Akt and AMPK act antagonistically to regulate mTOR signaling through inhibitory and activating phosphorylation of TSC2, respectively [33]. The Akt pathway is one of the most commonly affected pathways in cancer, with active PI3K/Akt signaling leading to excessive cell growth and proliferation [34,35]. Inhibition of this pathway by targeting mTOR with agents such as rapamycin is effective in some cancer types [36]. In a recent phase II trial in bone and soft tissue sarcomas, inhibition of mTOR with ridaforolimus resulted in better progression-free survival [13]. Inhibiting mTOR can, however, also activate a strong negative feedback loop from S6K1 to enhance Akt signaling [34,36]. It may, therefore, be more effective to inhibit Akt itself. Inhibition of Akt was recently tested in a panel of xenografts of different pediatric cancers, and was most effective in osteosarcoma, with significant differences in event-free survival in 6/6 xenografts [19]. In addition, AMPK activators suppress growth of cell lines of various tumor types [37].

We treated osteosarcoma cell lines with the allosteric Akt inhibitor MK-2206 (Selleck Chemicals LLC, Houston, TX). Inhibition of proliferation was dose-dependent in U-2 OS (IC_50_ 2.5 μM), but not in 143B (Figure 6). Important to note is that active Akt signaling can be detected by kinome profiling in this cell line, but this does not necessarily imply that this pathway can also be fully inhibited, for example in the case that downstream actors in the same pathway cause a survival benefit for the cell line. As 143B is derived from the HOS cell line with a *KRAS* oncogenic transformation, we determined inhibitory effects of MK-2206 on HOS as well. HOS responded to MK-2206 in a similar manner as U-2 OS (IC_50_ 2.6 μM). This suggests that constitutive Ras/Raf/ERK signaling causes insensitivity to inhibition of the Akt pathway to MK-2206. Kinome profiling of cells treated with MK-2206 resulted in different phosphorylation patterns in 143B and U-2 OS of peptides of molecules in the PI3K/Akt pathway (Figure 7). Differences between these cell lines were found in BAD Ser-99, of which phosphorylation was inhibited after treatment with MK-2206 in the responsive cell line U-2 OS, but stimulated in 143B, and in BAD Ser-118, where an opposite pattern was detected. BAD Ser-99 is the major site of Akt phosphorylation, while Ser-118 is the major site of PKA phosphorylation [38]. Opposite patterns were also detected for TP53 Thr-18 and CDKN1A Thr-145/Ser-146, of which CDKN1A Thr-145 can also be directly phosphorylated by Akt. These results suggest that activity of other kinases may be affected by inhibition of Akt using MK-2206, or by MK-2206 itself. This depends on the cellular context, as we otherwise would not have expected to detect any differences in a paired analysis for the different conditions in each cell type.

An important finding of our studies is that the PI3K/Akt and AMPK signaling pathways were detected with kinome profiling, while mRNA expression profiling did not result in the identification of these pathways. This suggests that in osteosarcoma, these pathways are regulated by phosphorylation rather than by transcriptional activity. Copy number and mRNA expression levels of Akt family members and their upstream players did not provide us with a possible mechanism for elevated Akt activity, although *PTEN* showed lower, but not significantly lower, gene expression levels in both cell lines as compared with the two MSC controls (*data not shown*). Gene expression and protein synthesis imply a long time commitment of a cell to potential activation of its synthesized proteins. Phosphorylation, on the other hand, provides a very rapid way to mobilize extra catalytic power for a short time, and allows fine-tuning of the activation of a pathway to the needs of a cell. This difference in time scale emphasizes the importance of applying different platforms for the analysis of a complex tumor as high-grade osteosarcoma.

## Conclusions

In summary, this study shows that genomic stability pathways are deregulated on both mRNA and kinome levels, with most significantly affected genes being upregulated and/or phosphorylated. Akt was detected as most probably overactive in osteosarcoma, as downstream peptides were hyperphosphorylated as compared with MSCs. Akt inhibitor MK-2206 could inhibit 2/3 osteosarcoma cell lines. Based on these results, we conclude that attenuating the PI3K/Akt/mTOR pathway may be effective in a subset of osteosarcomas.

### Description of additional files

The following additional files are available with the online version of this paper. Additional file 1 (.xls) includes the latest genotyping results of cell lines 143B and U2OS. Additional file 2 (.pdf) is a figure depicting unsupervised clustering of gene expression data. Additional file 3 (.pdf) is a figure showing differentially expressed genes in osteosarcoma cell lines versus control cell cultures. Additional file 4 (.pdf) depicts unsupervised clustering of all genes present in the significantly affected pathways determined by IPA analysis. Additional file 5 (.pdf) depicts Kaplan-Meier analysis of the different clusters detected in Additional file 4. Additional file 6 (.xls) is a table including results from the transcription factor activity prediction analysis in IPA. Additional file 7 (.pdf) is a Venn diagram showing significantly differentially phosphorylated peptides over time. Additional file 8 (.pdf) shows unsupervised clustering of technical replicates used in the kinome profiling experiment. Additional file 9 (.pdf) illustrates significant differential phosphorylation in the AMPK signaling pathway. Additional file 10 (.pdf) depicts distances between kinome profiling data of treated and untreated osteosarcoma cells using unsupervised clustering.

## Abbreviations

ALL: Acute lymphoblastic leukemia; DE: Differentially expressed; FDR: False discovery rate; adjP: FDR adjusted P-value; IC50: Half maximal inhibitory concentration; IPA: Ingenuity pathways analysis; logFC: Log fold change; MSC: Mesenchymal stem cell; Ser/Thr: Serine/threonine.

## Competing interests

Riet Hilhorst and Monique Mommersteeg are PamGene International B.V. employees. The other authors declare that they have no conflict of interests.

## Authors’ contributions

MLK performed all bioinformatics analyses and wrote the manuscript. RH and MM performed kinome profiling experiments. BEWMA and MLK performed inhibition studies. EPB and MS were involved in collection of cell line data. MLK, AMC, PCWH, RH, and HB designed the study. All authors read and approved the final version of the manuscript.

## Pre-publication history

The pre-publication history for this paper can be accessed here:

http://www.biomedcentral.com/1755-8794/7/4/prepub

## Supplementary Material

Additional file 1 Cell line identification of 143B and U2OS. Click here for file

Additional file 2 **Unsupervised clustering of gene expression data.** Unsupervised hierarchical clustering of mRNA expression data of osteosarcoma cell lines (black), MSCs (dark gray), and osteoblasts (light gray), on the 1,000 probes with highest variability in expression. Cell lines and controls cluster separately. Red: upregulation, green: downregulation. Click here for file

Additional file 3 **Genome-wide gene expression analysis.** MA plots of *A* osteosarcoma cell lines vs MSCs and *B* vs osteoblasts (OB). For each probe, log-intensity ratios (M) are plotted against log-intensity averages (A). Probes with adjusted *P*-values < 0.05 are shown in orange, while probes with adjusted *P*-values < 0.0001 are shown in red. Probes that do not show significant differential expression are depicted in black. Click here for file

Additional file 4 **Unsupervised hierarchical clustering on expression of genes in significantly affected pathways.** Hierarchical clustering of osteosarcoma cell line data (black), control cell lines (MSC: dark gray, osteoblast: light gray), and data from osteosarcoma biopsies (blue) on mRNA expression levels of all DE genes present in the 17 significantly affected pathways as determined by IPA. The different clusters selected for Kaplan-Meier analysis are shown in the upper dendrogram in different shades of blue, corresponding to the legend of Additional file 5. Red: upregulation, green: downregulation. Click here for file

Additional file 5 **Kaplan-Meier analysis of different clusters based on expression of genes in the significantly affected pathways.** Kaplan-Meier metastasis-free survival analysis on data obtained from patient biopsies which clustered with osteosarcoma cell lines, biopsies clustering with control cell lines, and an intermediate group, based on gene expression of genes all present in the 17 significantly affected pathways (as in Additional file 4). Log-rank test for trend, *P* = 0.049. Click here for file

Additional file 6 **Transcription factor analysis.** Results from the transcription factor activity prediction analysis in IPA, showing, for each transcription regulator the molecular type, the logFC of expression of the transcription factor itself, the predicted activation state (Activated/Inhibited), the regulation z-score, p-value, and the target molecules present in the dataset. Click here for file

Additional file 7 **Comparison of peptide phosphorylation at different time points.***LIMMA* analyses were performed on different time points, ranging from 0 to 60 minutes of incubation with cell lysates. Venn diagrams show overlap of significantly differentially phosphorylated peptides between the consecutive time points. Click here for file

Additional file 8 **Unsupervised hierarchical clustering of the technical replicates in kinome profiling.** Unsupervised hierarchical clustering on data from all technical replicates that were used for averaging the kinome profiling data. This clustering was performed on the significantly differentially phosphorylated peptides that were returned by a LIMMA analysis on the averages of the technical replicates, as depicted in Figure 3 of the manuscript. Peptides are sorted on logFC, from lower phosphorylation to higher phosphorylation in osteosarcoma cell lines. Orange: higher phosphorylation levels, blue: lower phosphorylation levels. Click here for file

Additional file 9 **AMPK signaling pathway.** The AMPK signaling pathway in IPA. Blue: significantly lower, orange: significantly higher phosphorylation in osteosarcoma cell lines, gray, no significant difference in phosphorylation, white: no phosphorylation sites of the particular protein on the PamGene Ser/Thr chip. Blue lines indicate known downstream phosphorylation by the upstream kinase. Click here for file

Additional file 10 **Distances between the kinome profiling data of cells treated with MK-2206.** Unsupervised hierarchical clustering depicting the distances between data obtained from kinome profiling of cells treated with different concentrations of MK-2206 and for different time intervals. 1_30: treatment of 30 min with 1 μM of MK-2206, etc. Click here for file
