# Supplementary material for: Kinome and mRNA expression profiling of high-grade osteosarcoma cell lines implies Akt signaling as possible target for therapy
Source: BMC Med Genomics. 2014 Jan 21;7:4. doi: 10.1186/1755-8794-7-4 (PMC3932036; doi:10.1186/1755-8794-7-4)
Supplement: Additional file 2 — Unsupervised clustering of gene expression data. Unsupervised hierarchical clustering of mRNA expression data of osteosarcoma cell lines (black), MSCs (dark gray), and osteoblasts (light gray), on the 1,000 probes with highest variability in expression. Cell lines and controls cluster separately. Red: upregulation, green: downregulation. [file 1755-8794-7-4-S2.pdf]

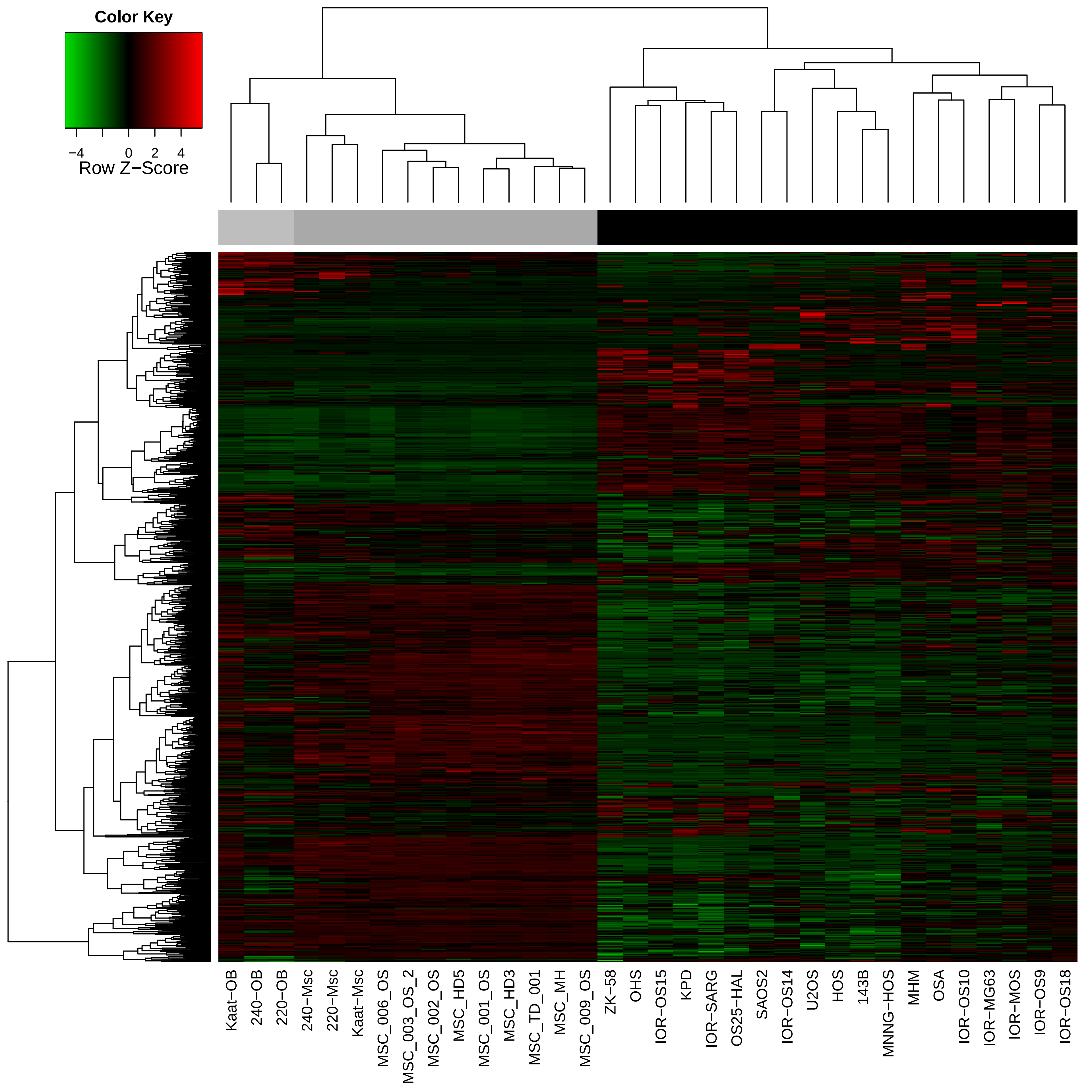
Color Key
−4
0
2
4
Row Z−Score
Kaat−OB
240−OB
220−OB
240−Msc
220−Msc
Kaat−Msc
MSC_006_OS
MSC_003_OS_2
MSC_002_OS
MSC_HD5
MSC_001_OS
MSC_HD3
MSC_TD_001
MSC_MH
MSC_009_OS
ZK−58
OHS
IOR−OS15
KPD
IOR−SARG
OS25−HAL
SAOS2
IOR−OS14
U2OS
HOS
143B
MNNG−HOS
MHM
OSA
IOR−OS10
IOR−MG63
IOR−MOS
IOR−OS9
IOR−OS18
