# Supplementary figures and images for: Kinome and mRNA expression profiling of high-grade osteosarcoma cell lines implies Akt signaling as possible target for therapy
Source: BMC Med Genomics. 2014 Jan 21;7:4. doi: 10.1186/1755-8794-7-4 (PMC3932036; doi:10.1186/1755-8794-7-4)

**OScellvsMSC**

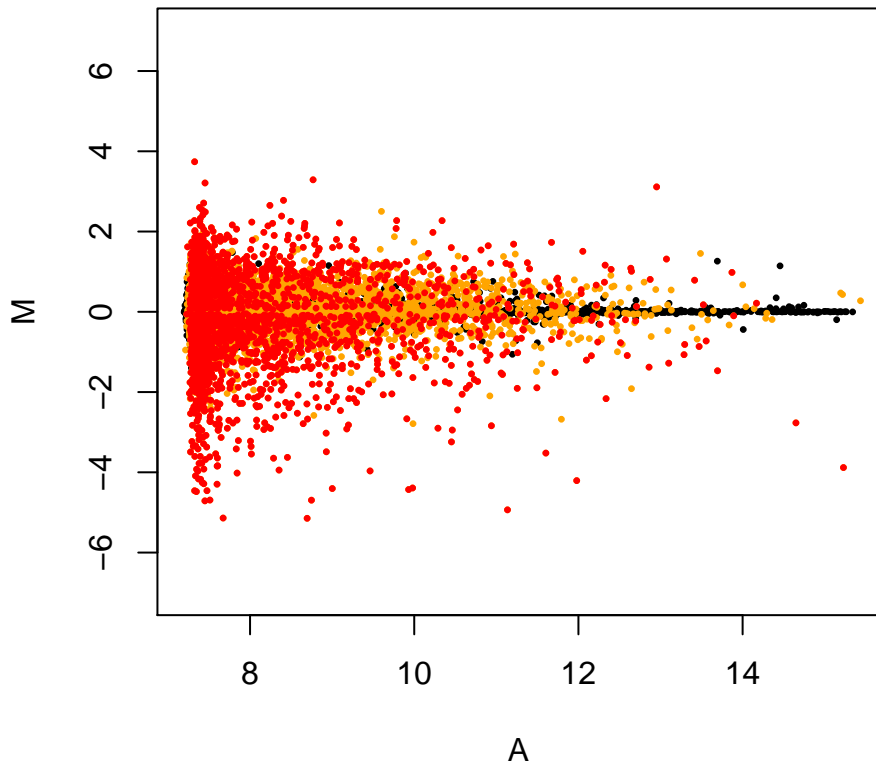

**OScellvsOB**

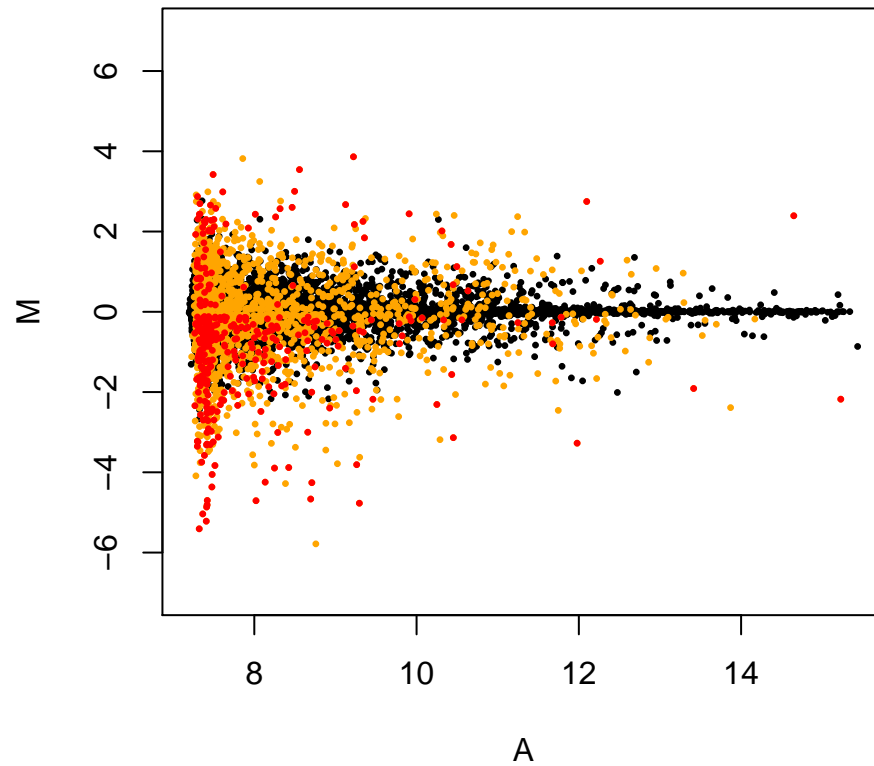

Supplement: Additional file 3 — Genome-wide gene expression analysis. MA plots of A osteosarcoma cell lines vs MSCs and B vs osteoblasts (OB). For each probe, log-intensity ratios (M) are plotted against log-intensity averages (A). Probes with adjusted P-values < 0.05 are shown in orange, while probes with adjusted P-values < 0.0001 are shown in red. Probes that do not show significant differential expression are depicted in black. [file 1755-8794-7-4-S3.pdf]

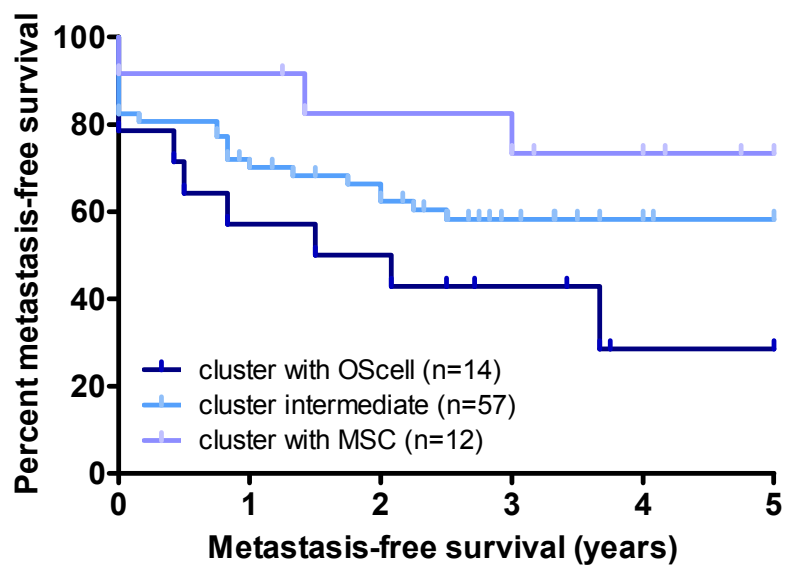

Figure 3

Supplement: Additional file 5 — Kaplan-Meier analysis of different clusters based on expression of genes in the significantly affected pathways. Kaplan-Meier metastasis-free survival analysis on data obtained from patient biopsies which clustered with osteosarcoma cell lines, biopsies clustering with control cell lines, and an intermediate group, based on gene expression of genes all present in the 17 significantly affected pathways (as in Additional file 4). Log-rank test for trend, P = 0.049. [file 1755-8794-7-4-S5.pdf]

40 min

20 min

60 min

0 min

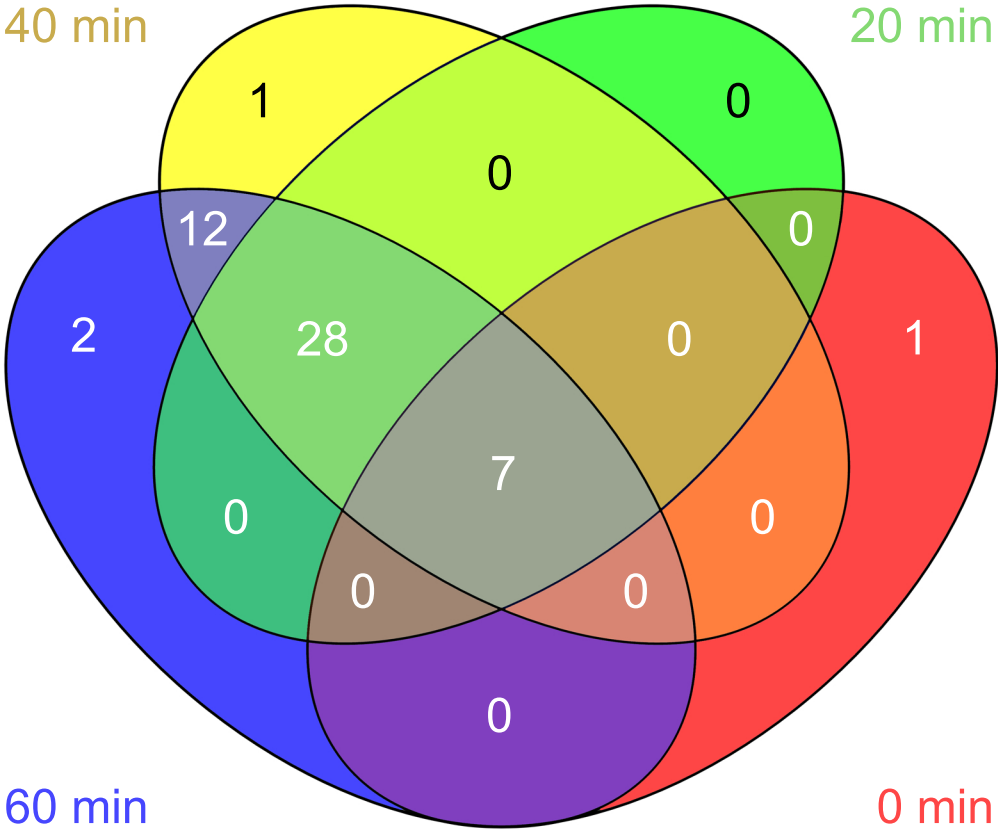

Supplement: Additional file 7 — Comparison of peptide phosphorylation at different time points.LIMMA analyses were performed on different time points, ranging from 0 to 60 minutes of incubation with cell lysates. Venn diagrams show overlap of significantly differentially phosphorylated peptides between the consecutive time points. [file 1755-8794-7-4-S7.pdf]

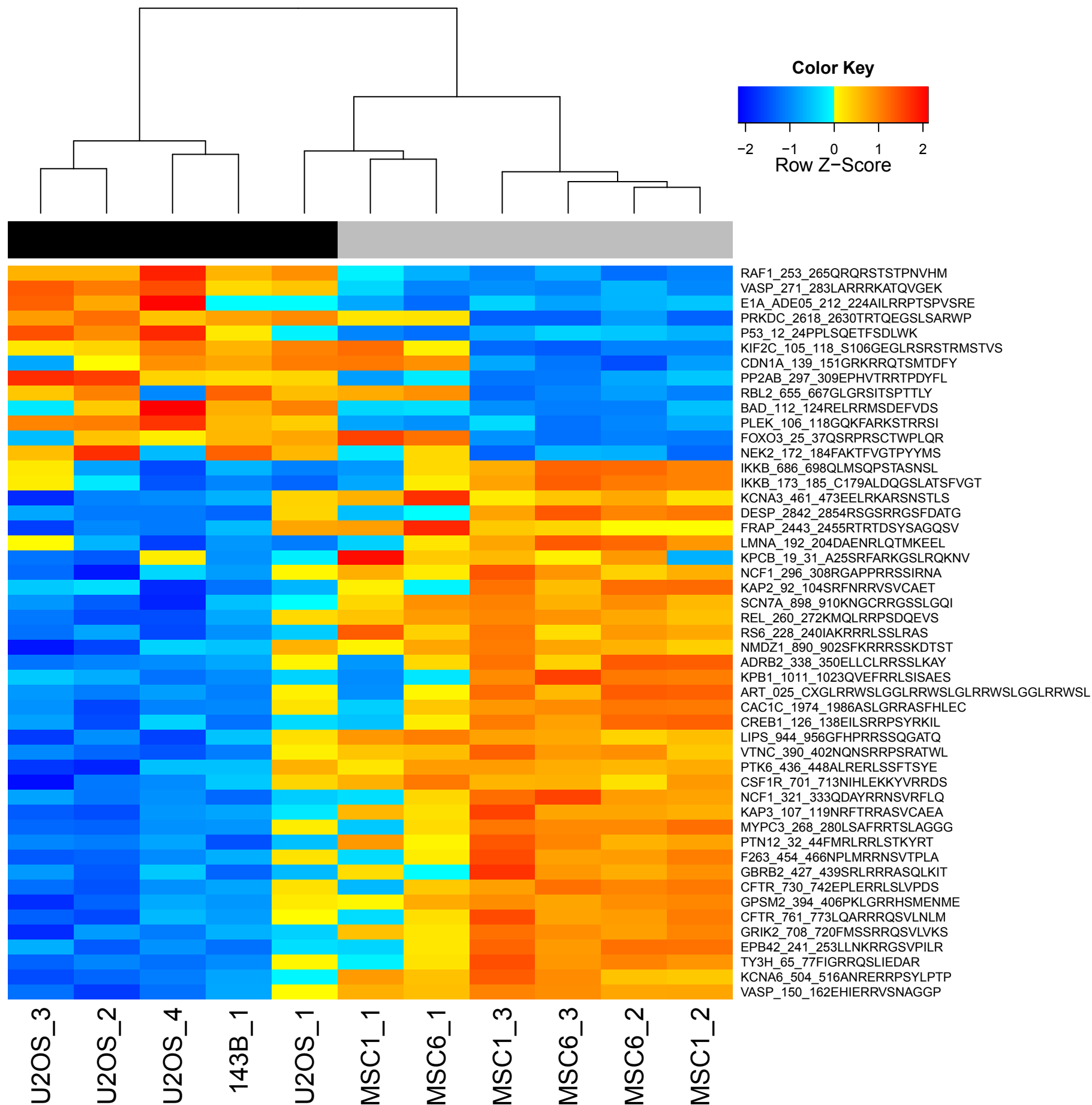

Supplement: Additional file 8 — Unsupervised hierarchical clustering of the technical replicates in kinome profiling. Unsupervised hierarchical clustering on data from all technical replicates that were used for averaging the kinome profiling data. This clustering was performed on the significantly differentially phosphorylated peptides that were returned by a LIMMA analysis on the averages of the technical replicates, as depicted in Figure 3 of the manuscript. Peptides are sorted on logFC, from lower phosphorylation to higher phosphorylation in osteosarcoma cell lines. Orange: higher phosphorylation levels, blue: lower phosphorylation levels. [file 1755-8794-7-4-S8.pdf]

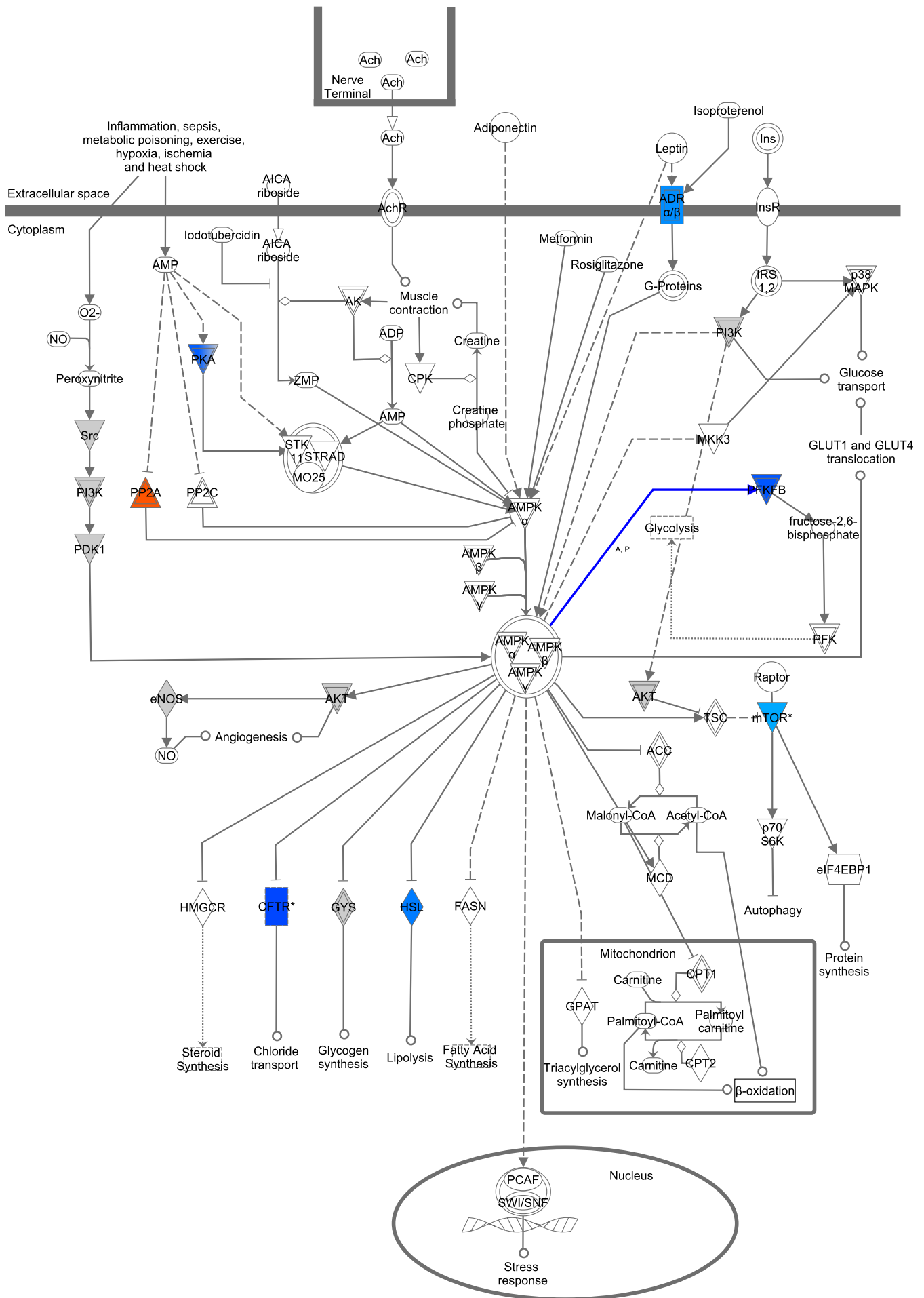

Supplement: Additional file 9 — AMPK signaling pathway. The AMPK signaling pathway in IPA. Blue: significantly lower, orange: significantly higher phosphorylation in osteosarcoma cell lines, gray, no significant difference in phosphorylation, white: no phosphorylation sites of the particular protein on the PamGene Ser/Thr chip. Blue lines indicate known downstream phosphorylation by the upstream kinase. [file 1755-8794-7-4-S9.pdf]

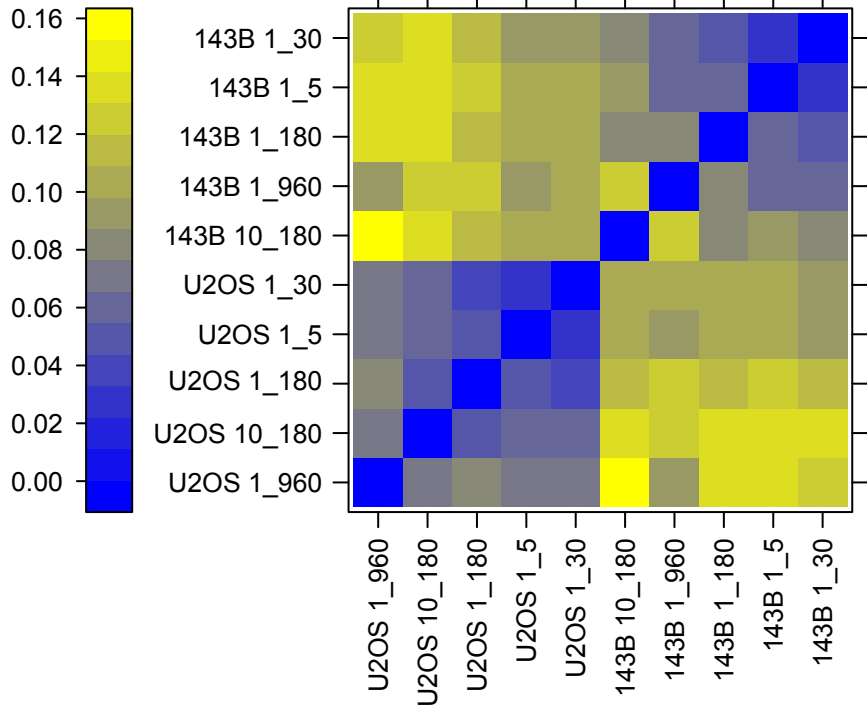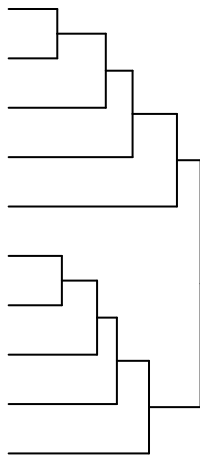

Supplement: Additional file 10 — Distances between the kinome profiling data of cells treated with MK-2206. Unsupervised hierarchical clustering depicting the distances between data obtained from kinome profiling of cells treated with different concentrations of MK-2206 and for different time intervals. 1_30: treatment of 30 min with 1 μM of MK-2206, etc. [file 1755-8794-7-4-S10.pdf]
