# Supplementary material for: Kinome and mRNA expression profiling of high-grade osteosarcoma cell lines implies Akt signaling as possible target for therapy
Source: BMC Med Genomics. 2014 Jan 21;7:4. doi: 10.1186/1755-8794-7-4 (PMC3932036; doi:10.1186/1755-8794-7-4)
Supplement: Additional file 4 — Unsupervised hierarchical clustering on expression of genes in significantly affected pathways. Hierarchical clustering of osteosarcoma cell line data (black), control cell lines (MSC: dark gray, osteoblast: light gray), and data from osteosarcoma biopsies (blue) on mRNA expression levels of all DE genes present in the 17 significantly affected pathways as determined by IPA. The different clusters selected for Kaplan-Meier analysis are shown in the upper dendrogram in different shades of blue, corresponding to the legend of Additional file 5. Red: upregulation, green: downregulation. [file 1755-8794-7-4-S4.pdf]

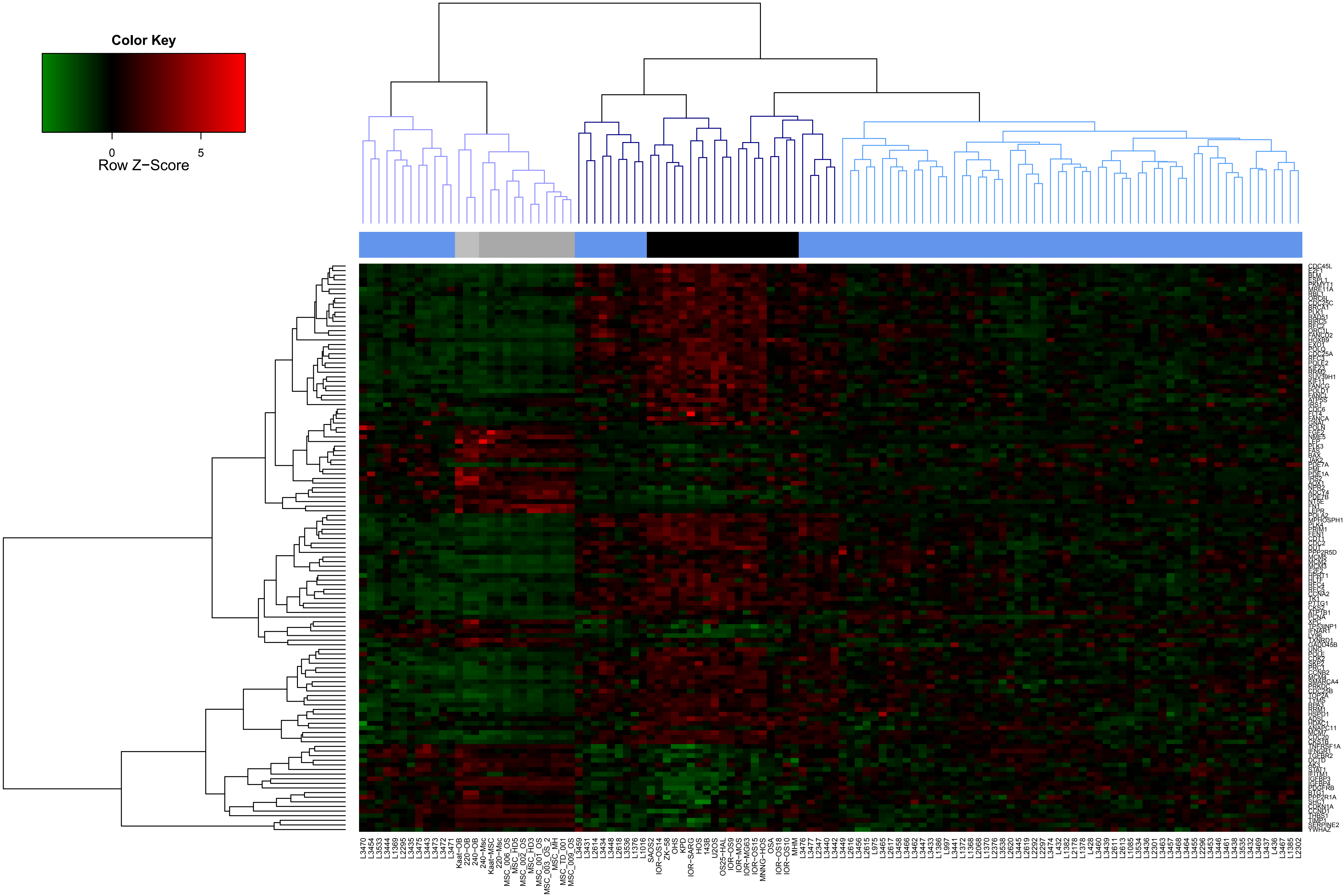
Color Key
0
5
Row Z−Score
L3470
L3454
L3533
L3444
L1369
L2295
L3435
L3475
L3443
L3473
L3472
L3471
Kaat−OB
220−OB
240−OB
240−Msc
Kaat−MSC
220−Msc
MSC_006_OS
MSC_HD5
MSC_002_OS
MSC_HD3
MSC_001_OS
MSC_003_OS_2
MSC_MH
MSC_TD_001
MSC_009_OS
L3459
L3431
L2614
L3434
L3448
L2618
L3536
L1376
L1016
SAOS2
IOR−OS14
ZK−58
OHS
KPD
IOR−SARG
HOS
143B
U2OS
OS25−HAL
IOR−OS9
IOR−MG63
IOR−OS15
MNNG−HOS
OSA
IOR−OS18
IOR−OS10
MHM
L3476
L2347
L3440
L3442
L3449
L2616
L3456
L2615
L975
L3465
L2617
L3458
L3466
L3462
L3447
L3433
L1386
L997
L3441
L1372
L1368
L2068
L1370
L2376
L3538
L2620
L3445
L2619
L2292
L2297
L3474
L432
L1382
L2178
L1378
L428
L3460
L3439
L2611
L2613
L1085
L3534
L3436
L2301
L3463
L3457
L3468
L3464
L3455
L2296
L3453
L3446
L3461
L3438
L3535
L3432
L3469
L3437
L436
L3467
L1385
L2302
